# Development and Preliminary Usability Evaluation of a Somatosensory Square Dance System for Older Chinese Persons: Mixed Methods Study

**DOI:** 10.2196/16000

**Published:** 2020-05-28

**Authors:** Chin-Wen Yu, Pei-Luen Patrick Rau, Xueqian Liu

**Affiliations:** 1 Department of Industrial Engineering Tsinghua University Beijing China

**Keywords:** older persons, chinese square dance, somatosensory games, somatosensory square dance system, physical exercise

## Abstract

**Background:**

Chinese square dancing, known as guangchang wu in Chinese, is a well-known public fitness activity that provides an entertaining way for older Chinese women to improve their flexibility, lower extremity strength, overall coordination, and balance. However, injuries, noise conflicts, and lack of space are challenging aspects of this activity. Somatosensory games (SG) are an increasingly popular physical fitness approach to enhance the selective attention of older persons with indoor engagement and exercises.

**Objective:**

The objectives of this study were to develop a newly designed somatosensory square dance system for older Chinese people and to evaluate its usability.

**Methods:**

This is a mixed methods study. The newly designed somatosensory square dance system is a somatic training tool that provides adequate Chinese square dance fitness training based on Laban Movement Analysis (LMA) and design guidelines established in a previous stage. The usability evaluation involved a questionnaire and interviews. Twelve participants were interviewed before and after experiencing the 15-minute dancing and learning process within the program. In addition, participants scored their experience satisfaction in psychological, physiological, and relaxation sections on a scale of 1 to 5 using a questionnaire. Qualitative content analysis and quantitative analysis of the satisfaction scores supported understanding of usability problems.

**Results:**

Based on the interview results, 6/12 (50%) of the participants thought the system could help them correct their dancing movements indoors without being affected by poor outdoor weather. Among the participants, 3/12 (25%) indicated that this indoor system could enable them to enjoy fitness activities in a private space. Moreover, 3/12 participants (25%) stated that this system could promote family relationships by providing easy dancing movements. All participants were highly satisfied with the relaxation aspect of the system (4.45/5). The participants were all psychologically satisfied and interested in the novel features of the system, with an average score of 4.16/5. Physiologically, participants affirmed that the system could help them maintain good health (4.91/5).

**Conclusions:**

The results of this study suggest that the somatosensory square dance system can be used as an indoor fitness tool to improve older Chinese square dancers’ health conditions with reasonable dance training. Noise and space conflicts can be addressed. The Laban Elated Square Dance system, which was modified by therapists based on LMA and square dance design guidelines, was highly approved by dancers because it decreased the possibility of injuries, falls, and joint damage by considering the physical and psychological difficulties of older persons. Different features will be considered in the next stage, such as greater selection of exercises and difficulty level settings. Users’ social needs will also be explored in subsequent stages.

## Introduction

### Background

In recent years, countless “dancing grannies” have been observed dancing in groups to loud and intense music in tranquil neighborhoods across China; this dance form is known as Chinese square dance (guangchang wu in Chinese). It is generally believed that Chinese square dance is an extension of line dancing, which was introduced in China around 2004 [1]. The year 2015 is called the first year of Chinese square dance because it was performed on stage at the Chinese Spring Festival Gala. In the same year, 2 government agencies, the State General Administration of Sports and the Ministry of Culture, announced the development of 12 model square dancing routines; instructors were hired and trained to introduce these routines around the country. Thereafter, square dancing was quickly acculturated by the integration of Chinese dance styles and music. For several different reasons, including fitness, socialization, and governmental promotion, Chinese square dance has become one of the most popular exercise formats in China. Due to its low cost and ease of participation, Chinese square dance is a popular pastime. According to a report by the Economist Corporate Network in 2017 [2], the size of China's sports fitness market in 2016 was close to 1.5 trillion yuan, and over 400 million Chinese people exercise regularly. Chinese square dance represents 25% of this exercise. Chinese square dance differs from traditional square dancing, in which 4 couples are arranged in a square with one couple on each side facing the middle of the square; Chinese square dance groups consist of older or retired women who spontaneously congregate in the early morning and evening at any time of year in parks, public squares, or any place they can find. Chinese square dance represents the collective aspect of Chinese culture. With lively step movements and strong rhythm, it has already become the most popular fitness approach among retired, middle-aged, and older women. It is also an effective way to maintain mental awareness; Chinese square dance can improve memory and can even delay some of the negative traits of diseases such as Alzheimer disease and other forms of dementia, which are currently becoming more frequent as people live longer. According to the data in the 2017 China Square Dance White Paper [3], people aged 18 to 52 years are the main force of Chinese square dance, with a proportion of 71%; only 27% of dancers are more than 52 years old. East China has the highest number of dancers in Generation Y, with a proportion as high as 43% or more. However, it has been reported that older people can be easily injured by falling during square dance exercise due to lack of instruction and professional guidance. When middle-aged people dance, they perform many movements using joints and muscles that are not highly stretched. Performing these movements is harmful to the body. Additionally, square dancers come into conflict with residents of the areas that surround their preferred spaces. Many younger workers have complained that the noise pollution prevents them or their families from obtaining needed rest, especially if different groups of dancers increase the volume of their music to compete with each other. To date, there is little research or study on designing indoor Chinese square dance training systems or software to prevent injury and provide fewer interruptions for other people.

### Literature Review

According to previous studies, exercise and activity significantly reduce the risk of cardiovascular disease, stroke, hip fracture, osteoporosis, and falls [4-6]. Dance-based therapy has been found to improve balance and locomotion [7-9], lower body strength, flexibility, and endurance [7,10-12]; it also enhances motivation and improves psychological well-being [13]. Studies have demonstrated the value of square dance and its impact on the physiological, psychological, and social needs of individuals. Square dance has been found to could reduce the rate of decline of bone mineral density, improve balance, and decrease the risk of falls in postmenopausal women [14]. One study compared the influences of Tai Chi exercise and square dancing on individuals’ physical indices; they found that square dancing had a more significant effect on waist-hip ratio changes [15]. In one study, it was discovered that participants’ chest circumference increased gradually during square dance exercise and that their waist and hip circumference and waist-hip ratio both gradually decreased [16]. Evidence has been reported that square dancing can effectively reduce the blood glucose and HbA1c levels of patients with type 2 diabetes [17]. Square dance exercise could significantly improve the happiness index of older women, which was highest in a square dance exercise group [18]. On the other hand, this form of exercise has negative impacts, such as falls and injuries, and environmental problems also occur. One study reported that 66.6% of older persons encountered different levels of injuries in square dance fitness due to lack of risk awareness [19]. Another study noted risk factors such as lack of facilities, lack of tutoring and professional knowledge, and inadequate attention [20].

Laban Movement Analysis (LMA) is an analysis tool that is used to observe, describe, and record various human actions during dancing movements [21]. LMA enables observation and analysis of body movements, and it has been applied in various fields, including early childhood development, enterprise management, and psychiatric treatment, as well as in academic applications such as psychology, kinematics, sociology, and criminology [22]. Laban proposed that effort is the most basic tool for human movement. A process was established to record the elements of effort, which consist of four motion factors: space, weight, time, and flow. LMA experts were recruited to analyze different versions of a dance according to the space, weight, and time factors [23]. They discovered that the official version of the dance involved a higher proportion of strong effort, while two other versions involved lighter and gentler movements; strong effort was more likely to create feelings of stress and impatience in the human body. The experts concluded that the official version of the dance was undesirable because it required a large amount of strong effort; meanwhile, movements requiring sustained effort could effectively train coordination. Fitness guidelines for square dancers based on LMA to prevent falls and injuries were designed, and the researchers concluded that when the space factor was generalized as a direct effort, the movements were easy to learn. The recommended ratio for strong and light effort movements was 3:7, which could pacify the dancers’ emotion and train the muscle strength of their lower extremities. Definitions and directions of the four motion factors are provided in Tables 1 and 2.

In motion sensing technology, the human body functions as a controller. Users do not need to touch the equipment directly; their body language is located by the equipment, which receives instructions accurately. Somatosensory sensation can also be called somatic sensation; it comprises the senses of touch, pressure, temperature, pain, and body feel. Somatosensory operation is characterized by intuition, fun, and interactivity. Compared to the operation of previous controllers, a somatosensory system can be easily learned by waving and moving the hands and feet, by which users can be fully engaged and immersed. Somatosensory technology was initially used in the game industry to provide indoor entertainment. In recent years, its scope of application has gradually expanded to athlete monitoring, health care, virtual dressing rooms, rehabilitation, etc. Serious games are promising as an effective, lasting, low-impedance method to improve the social, cognitive, sensory, and emotional functions of older people [24]. Users can simultaneously realize the unified benefit of game playing, sports simulation, education, exercise, disease prevention, and rehabilitation [25].

In recent years, an interactive platform with Kinect somatosensory equipment (Microsoft Corporation) was developed that can help people with disabilities undergo treatment and rehabilitation at home [26]. Gutierrez et al [27] studied 2 groups of patients with multiple sclerosis; one group received traditional treatment, and the other received remote somatosensory treatment. After 10 weeks, the balance and posture control of the 2 groups of patients both showed significant improvement. One study applied Kinect to training exercises that were prescribed for older people; they found that the participants’ sense of balance improved, but their willingness to participate was not high because the levels of entertainment and interest were not as high as expected [28].

**Table 1:** Definitions of the four motion factors of Laban Movement Analysis.

Motion factor	Definition
Space	The degree of change between the movement and the sense of space (condensed or loose).
Weight	The degree of change of the center of gravity in motor performance (lifted up or falling).
Time	The degree of change of the sense of time.
Flow	The body flow state associated with individual muscle changes.

**Table 2:** Directions of the effort elements for the four motion factors of Laban Movement Analysis.

Effort element	Direction
Direct	Affirmative and targeted action execution with a simple path and clear starting and end points.
Indirect	Action execution is not clear, focal length is loose, and motion paths have many turning points.
Strong	Action does not necessarily decrease; strong performance of center motion with a large amount of resistance force.
Light	Light and soft performance; most actions defy gravity.
Sudden	Rapid changes in operation, inability to predict the next moment of action, anxious and uneasy performance.
Sustained	Actions change slowly, calmly, and continuously and are endless.
Bound	Physical manifestations of tension and muscle force. The operating state can be stopped at any time.
Free	Physical manifestations are relaxed, at ease, and unrestrained.

### Goals of This Study

In the field of Chinese square dance, few studies have focused on the development of somatosensory systems to improve training and address the problems of outdoor noise, venue availability, and privacy. In this study, we propose the following research questions: (1) How can we design and improve a more suitable Chinese square dance program using Laban Movement Analysis? To address existing problems such as injuries, falls, and lack of professional guidance, in this study, we propose a newly designed Laban Elated Square Dance system to maintain the flexibility, lower extremity muscle strength, and overall coordination and balance of older persons, thus reducing the negative physiological impacts of Chinese square dance. (2) How can we design an indoor somatosensory dance system to help resolve existing space constraints, public disturbance, and privacy issues? (3) How can we apply this somatosensory system to provide professional training instructions for older persons and incorporate this technology into family medical care?

In this study, we designed a somatosensory square dance system to provide suitable solutions for older persons with potential health problems (such as injuries and falls) and other problems (such as noise, lack of space, and privacy issues). We evaluated the usability and benefits of the Laban Elated Square Dance system and tested users’ satisfaction with its physiological and psychological impacts. Many previous studies have pointed out that the application of somatosensory interaction systems has great benefits in the field of rehabilitation; however, few studies have targeted the experience satisfaction of the general middle-aged and older population when using a somatosensory system indoors. This study also addressed users’ evaluations and definitions of the somatosensory Chinese square dance system.

## Methods

### Somatosensory Square Dance System Development

#### The Laban Elated Square Dance

**Figure 1:**
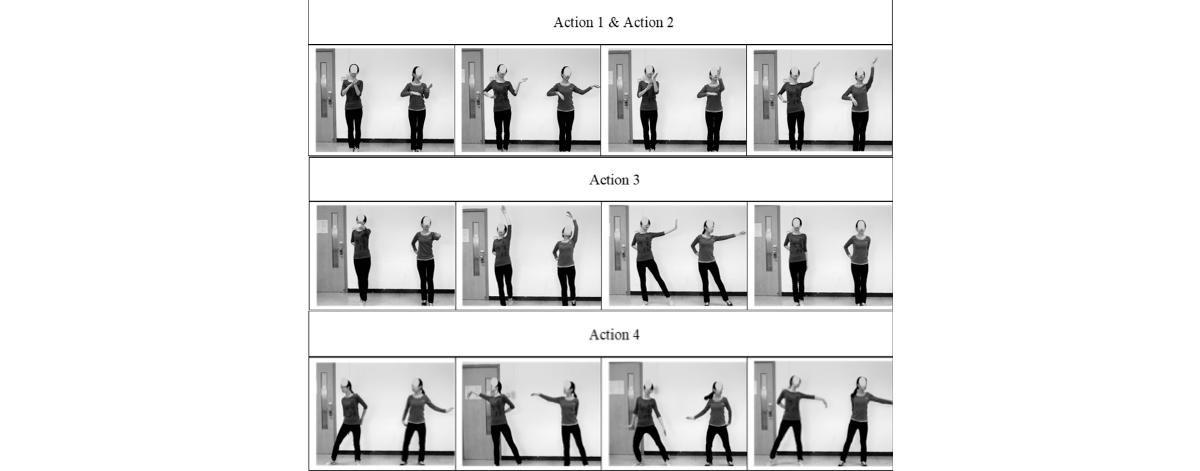
Actions 1-4 of the Laban Elated Square Dance.

In a previous fitness guideline paper, Yu, Rau, and Zhong [23] developed recommendations of fitness guidelines for rehabilitation therapists. Based on these guidelines, in this study, we choreographed a square dance that could aid balance training for middle-aged and older persons, called the Laban Elated Square Dance. The song “I Am From Prairie” has strong rhythmic melodies and was chosen as the background music. We designed 10 sets of movements with 16 steps in each set; dancers could repeat these movements several times during the song. Figure 1 describes the first four actions of the Laban Elated Square Dance.

Actions 1 and 2: Both hands are lifted to the chest, with palms lateral, right palm down and left palm up. The right elbow initially extends to the right and the left palm initially extends to the left until it reaches shoulder height/above shoulder height. In the opposite direction, the left and right hands are switched.

Action 3: On the first beat, the left hand extends forward at shoulder height, and the left toe taps forward; on the second beat, the left hand extends upward, in line with the body, and the left toe taps backward; on the third beat, the left hand at shoulder height extends to the left and the left toe taps to the left; on the fourth beat, the body returns to a standing position. The right arm is close to the back during the 4 beats. In the reverse direction, the left and right sides are switched.

Action 4: The arms swing back and forth behind the body, and the center of gravity shifts as the body swings; when shifting to the right, the right hand extends to the far end. The arms swing back and forth again, and when shifting to the left, the left hand extends to the far end.

Action 5: A cross step is taken to move forward, with the center of gravity on the front foot and the rear foot in the tiptoe position. The hands are held flat to the left at shoulder height. Afterward, on the left side, the front and rear legs exchange positions, with the hands at the right at shoulder height. A total of 2 steps forward are taken during the 4 beats.

Action 6: The right hand is placed on the back, with the left hand drawing a circle from front to rear, with the arm always extended, similar to stretching movements; the left toe is tapped behind for 1 beat, then is returned in 1 step to a position behind the right foot. When shifting to the right side, the left and right sides are switched. For 2 beats, the movement switches to the left, and for 2 beats, it switches to the right, while taking a total of 2 steps backward.

Action 7: Starting from the chest position, the left hand draws a semicircle parallel to the ground and is then raised. On the first 2 beats, the left foot steps a shoulder distance away. On the second 2 beats, the left foot moves to the tiptoe position behind the right foot. The right hand remains at shoulder height as at the beginning, with the right foot in a stationary state to maintain body balance.

Action 8: With wrists bent, on the first beat, the left elbow is raised to a high point and the right elbow is pressed down to a low point, with the left foot stepping a shoulder-width distance. On the second beat, the direction changes, and the right foot and the left are brought together. The above movements are 2-beat movements; after 4 beats, a total of 2 left steps are taken.

Action 9: The head movements are the same as in Action 8; the difference is that the movement is performed while standing still and circling but without moving to the left or right.

Action 10: The hands and arms are open to the sides, with the hands performing a wavelike flowing motion. As the left hand moves upward gently, the right hand is pulled down; at the same time, the lower limbs tread and step together.

**Figure 2:**
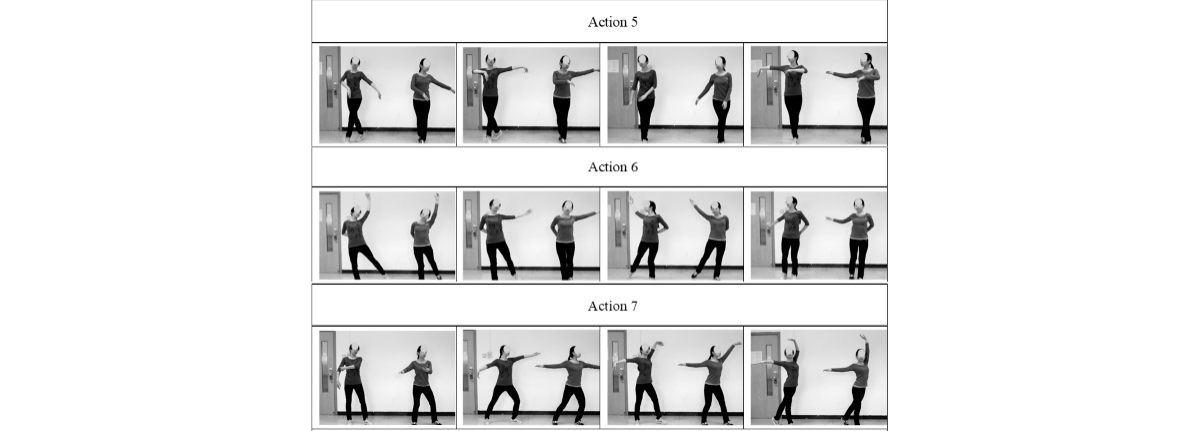
Actions 5-7 of the Laban Elated Square Dance.

**Figure 3:**
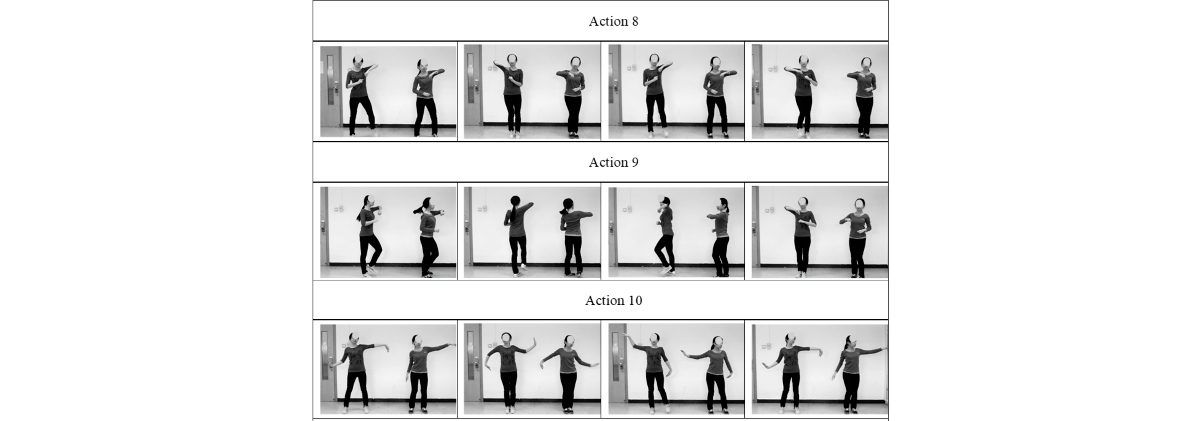
Actions 8-10 of the Laban Elated Square Dance.

Figure 2 describes actions 5-7 of the Laban Elated Square Dance. Figure 3 describes actions 8-10 of the Laban Elated Square Dance.

#### Development of the System

The indoor somatosensory square dance system used a Kinect 2 system to collect data and RGB images to track and identify human body movements; program coding of the system was also performed. Kinect 2 can detect 25 joints; however, it does not have a graphical interface. Therefore, we applied the Open Source Computer Vision Library with a computer and camera to help identify the participants and perform further image processing of the videos. The Kinect SDK 2.0 software development package was used in this system.

This system employed Visual Gesture Builder within the Kinect software to build the gesture identification database. The steps of the basic process used by Visual Gesture Builder to establish a custom gesture and enable the program to automatically identify it are as follows.

##### Step 1: Kinect Studio Records the Movement

**Figure 4:**
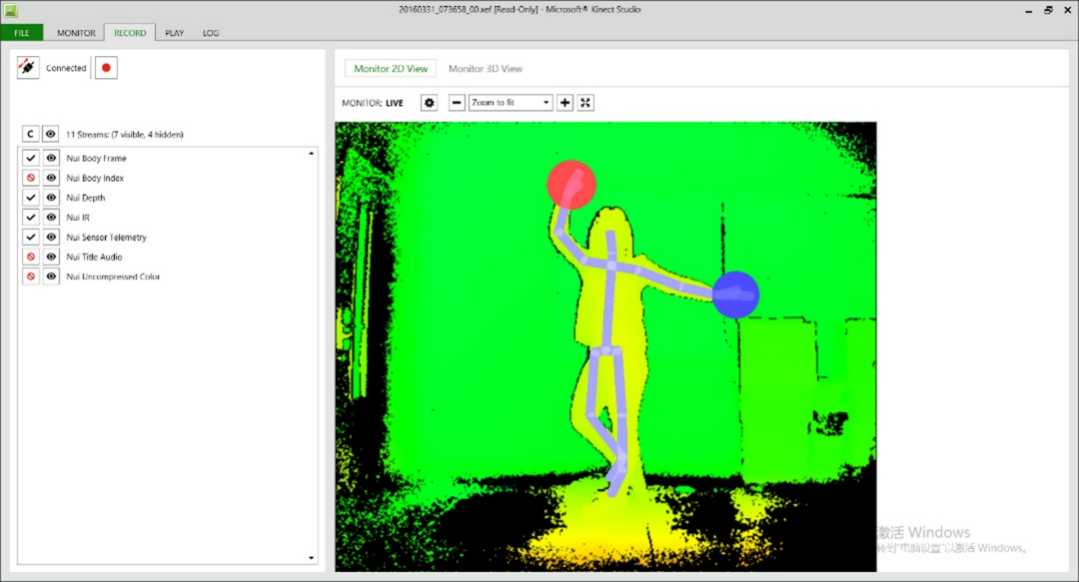
Recording interface of the Kinect Studio system.

Kinect Studio is a tool in the Kinect SDK 2.0 package that can be used to preview Kinect data; machine learning technology was applied here to recognize the repetitive dance movements in the video recording. The recording interface is shown in Figure 4.

##### Step 2: Build Individual Movement Through Visual Gesture Builder

**Figure 5:**
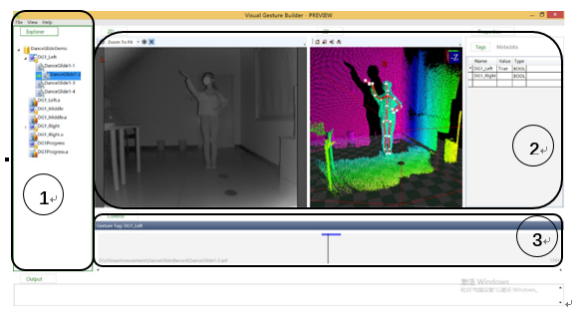
Video Gesture Builder interface for gesture labelling.

The recording profile was inserted into Visual Gesture Builder, and the movements were labeled manually. After all the movements which required evaluation were labeled, the gesture database was built. All the static posture and continuous movements were labeled. Figure 5 shows the Visual Gesture Builder interface used for gesture labeling.

**Figure 6:**
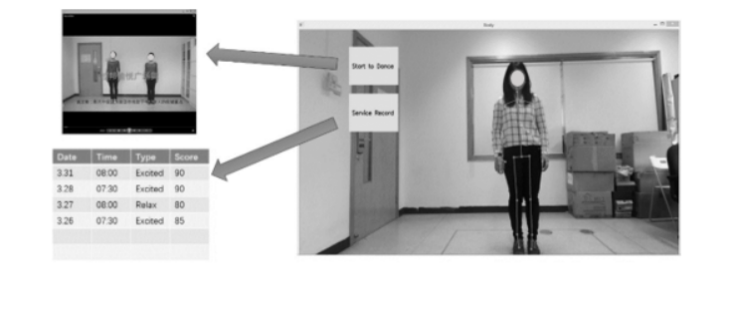
Interaction interface of the somatosensory square dance system.

The somatosensory square dance system was established for an older population; therefore, the visual interface was designed to be intuitive and simple. The platform consisted of a main interface and sub-interfaces that can interpret teaching and monitor practice records (Figure 6).

The system contained 80 separate dance movements; 20 points was the basis, and 1 point was added for each movement accomplished.

### Participants

We recruited 12 participants; 6 were older people (aged 55-68 years, mean age 60 years, SD 5.65) who were retired teachers at Tsinghua University, and 6 were younger people (aged 19-27 years, mean age 24.1 years, SD 2.71) who were undergraduate students from Tsinghua University. The young participants were recruited from the growing proportion of Generation Y, as mentioned in the Introduction [3].

### Procedures

Each participant was invited to the human-computer interaction laboratory at Tsinghua University. The overall experimental process lasted approximately 40 minutes for each participant. A single participant experienced the system each time, accompanied by a laboratory researcher. Before the start of the experiment, the researcher provided a basic introduction and explained the research purpose of the experiment as well as the rights and freedoms of the participants; the researcher assured the participants that all the information they provided was protected and ensured that the participants had no other questions or requests. This research complied with the American Psychological Association Code of Ethics and was approved by the Institutional Review Board at Institute of Human Factors and Ergonomics, Tsinghua University.

After the experiment began, the researcher interviewed the participant, asking questions about the participant’s basic information, past impressions, and experience of Chinese square dance. When experiencing the somatosensory system, every participant completed the Laban Elated Square Dance without time or frequency limits. If the participant wanted to stop, they could ask the researcher to stop. In the upper right corner of the instructional video, detailed guidance was provided for every movement regarding different body parts, such as coordinated training of the whole body, increased hip joint activity, or balance training. After experiencing the system, each participant was asked to provide experience satisfaction scale scores and to answer the researcher’s interview questions, such as the difference between outdoor and indoor Chinese square dance and their feelings about the system.

**Figure 7:**
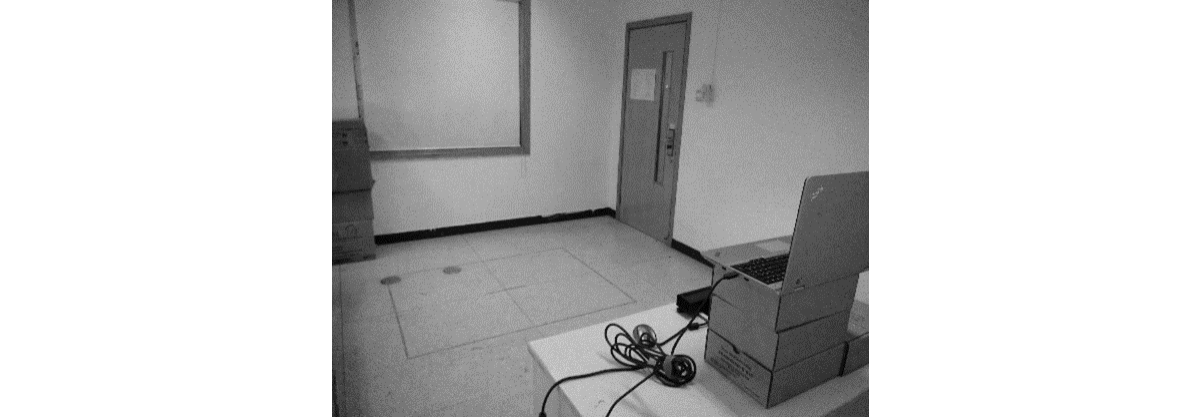
Setting of the experiment.

**Figure 8:**
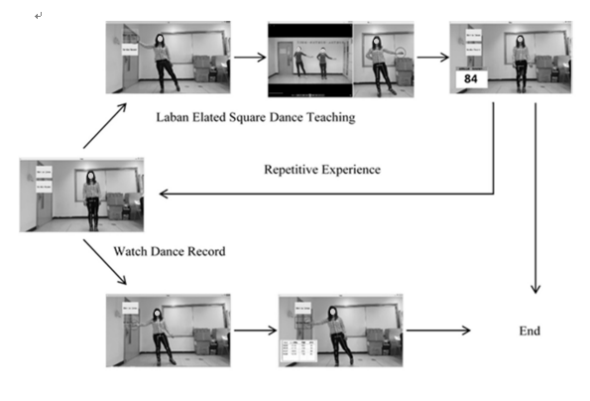
Practical experience of the indoor somatosensory square dance system.

The participants were instructed to stand in a rectangular area, with the initial standing point at the center. The equipment used was Kinect 2 for Windows and a computer. Figure 7 illustrates the setting in which the participants used this somatosensory square dance system. Figure 8 shows the practical routine the participants performed when experiencing the system.

### Measurements

The measurements used in the experiment include psychometric tools, such as a self-reported questionnaire and interviews, and objective behavioral measurements. The objective behavioral measurements, such as the degree of motion specification and the motion trajectory, were captured by the Kinect 2 system. The experiment was also recorded on video for further analysis of the comments of the participants. We interviewed the participants before the experiment to understand their impressions and past experience in square dance and after the experiment to determine their evaluation of the system and their feelings about it after experiencing it. The interview outline is shown in Textbox 1.

The questionnaire was based on the Leisure Satisfaction Scale designed by Beard and Ragheb [29]. In this study, the questionnaire was modified to contain three sections: psychological, physiological, and relaxation. These modifications were performed because the experience satisfaction degree in this study was based on the physical, psychological, and emotional satisfaction of the somatosensory system produced by the participants after experiencing it; therefore, only three parts of the scale were used. A Likert scale was applied to score the experience satisfaction in Table 3, from 1=“strongly disagree” to 5=“strongly agree.” All 12 participants answered the questionnaire. All statistical analyses were performed using Excel 2016 for Windows (Microsoft Corporation).

**Outline of interview questions before and after the participants’ experience with the somatosensory system.**

**Before the experience**

1. What is the definition of square dance?
2. What is the advantage of square dance? Or disadvantage?
3. Have you participated in square dance? What is the reason encouraging (or hindering) your continuous participation?
4. Have you experienced a somatosensory game before? How?

**After the experience**

1. What groups do you think this system is suitable for?
2. How does the somatosensory system square dance differ from outdoor square dance?
3. After using the somatosensory system, what would encourage or hinder your adoption of the system?
4. How did your body change after the fitness training with the somatosensory system?
5. What is your overall rating of the square dance somatosensory system?

**Table 3:** Psychological, physiological, and relaxation sections of the experience satisfaction scale in the questionnaire.

Section	Answer prompts
Psychological	It makes me feel interested.
	It makes me feel confident.
	It makes me feel a sense of accomplishment.
	It makes me try different techniques.
Physiological	It challenges fitness.
	It enhances physical ability.
	It increases my interest in sports.
	It keeps me healthy.
Relaxation	It can help me relax.
	It can help me release stress.
	It can make me joyful.
	It can help my emotional health.

## Results

### User Statistics

A summary of the demographic data and personal information of the participants is provided in Table 4.

### Interviews

Participants’ comments were collected and analyzed descriptively based on the interview outline. Based on their feedback, 5 responses were classified.

In response to question 1, “What kind of groups do you think this system is suitable for?”, 6/12 participants (50%) suggested that the user group could include older women with a fixed habit of outdoor square dance. Participants stated that with this system, they could correct and refine their skills; additionally, they could work out at home when weather conditions were poor. Additionally, 3/12 participants (25%) recommended that the user group could include people unfamiliar with square dance, such as young people and older men, who may be interested in square dance but would prefer a private space to learn; 3/12 participants (25%) recommended the system as a family game, since existing somatosensory games are more intense games. It was suggested that this system could be used as a substitute for television and could be a great style of entertainment.

In response to question 2, “What is the difference between the somatosensory system square dance and the outdoor square dance?”, 6/12 participants (50%) mentioned the advantages of social activity in traditional outdoor square dance, including greeting neighbors and interacting with teachers. Moreover, 3/12 (25%) mentioned environmental problem, such as air pollution and noise; 3/12 (25%) mentioned privacy problems, and they thought users could thoroughly enjoy themselves when using this system without judgment from others.

**Table 4:** Demographic and personal data of the participants (N=12).

Participant		Gender	Chinese square dance frequency	Duration of square dance activity (years)	Other fitness activities
**Younger participants (n=6)**					
	1	Female	2 times a week	N/A^a^	None
	2	Female	None	N/A	Running
	3	Female	None	N/A	None
	4	Female	None	N/A	Street dancing
	5	Female	None	N/A	None
	6	Male	3-5 times a week	N/A	None
**Older participants (n=6)**					
	7	Female	5 times a week	2	None
	8	Female	6 times a week	2.5	None
	9	Female	5 times a week	1	None
	10	Female	5 times a week	2	None
	11	Female	None	N/A	None
	12	Male	None	N/A	Running

^a^Not applicable.

In response to question 3, “After using the somatosensory system, what would encourage or hinder your adoption of the system?”, 7/12 participants (58%) mentioned the score feature; after learning the Laban Elated Square Dance, assessing their progress created more interest and confidence for the majority of young participants. Of the 12 participants, 2 (16.7%) mentioned directional confusion; because the teacher in the film danced in the opposite direction from reality, they became confused and uncertain. Additionally, 1/12 participants (8.3%) indicated that the button color was too bright to see, 1 participant (8.3%) felt it was interesting to see herself in the video, and 2 participants (17%) thought that if they could watch the video in the system before becoming familiar with the movement patterns, they could better understand their movement accuracy.

In response to question 4, “How did your body change after the fitness training with the somatosensory system?”, 4/12 participants (33%) mentioned that the design and arrangement met their physical needs, while 4 participants (33%) mentioned the professional aspect of the dance; they felt more secure because the dance was taught by a rehabilitation therapist. Additionally, 4/12 participants (33%) considered that they did not exercise enough and did not take fitness training into consideration.

In response to question 5, “What is your overall rating of the square dance somatosensory system?”, 2/6 older participants (33%) indicated that the somatosensory system was novel, and 3/6 older participants (50%) who usually participated in square dance gave some recommendations, such as interface design and increased functionality. They expressed interest in learning more physical information through new technology. Moreover, 2/6 younger participants (33%) suggested adding different movement difficulties and music choices to increase the exercise options.

Statistical data analyzed from the three different sections of the satisfaction scale questionnaire are shown in Table 5.

The relaxation section received the highest scores of the three scales, indicating that the users were more than satisfied; “It can help me relax” had the highest evaluation (mean 4.58). “It can help me release stress” received the lowest score; however, the overall scores all indicated high satisfaction, which indicates that the 12 participants generally believed that the somatosensory square dance system is relaxing, satisfying, and pleasant. The psychological scale was the second highest (mean 3.83). The average score was higher than normal, which is close to satisfaction. “It makes me feel interested” was the most common response. This indicates that the system is a novel type of activity for middle-aged and young people. The lowest score was for the physiological scale (mean 3.64). “It keeps me healthy” was the most common response in the physical scale. Most of the participants believed that continuous use of this system would definitely be helpful for body function.

**Table 5:** Statistical analysis of the participant responses to the satisfaction scale questionnaire (1=”strongly disagree” to 5=“strongly agree”).

Questionnaire section and prompt		Scale score, mean (SD)	
**Psychological satisfaction**			
	It makes me feel interested.		4.16 (0.71)
	It makes me feel confident.		3.83 (0.93)
	It makes me feel a sense of accomplishment.		3.67 (0.88)
	It makes me try different techniques.		3.67 (0.49)
**Physiological satisfaction**			
	It challenges fitness.		3.67 (0.77)
	It enhances physical ability.		3.58 (0.79)
	It increases my interest in sports.		3.41 (0.66)
	It keeps me healthy.		3.91 (0.79)
**Relaxation satisfaction**			
	It can help me relax.		4.58 (0.51)
	It can help me release stress.		4.33 (0.49)
	It can make me joyful.		4.41 (0.51)
	It can help my emotional health.		4.5 (0.52)

## Discussion

### Principal Findings

Based on Laban Movement Analysis (LMA) of previous Chinese square dance movements, in this study, we designed a Laban Elated Square Dance for middle-aged and older persons to enhance balance training. We developed an indoor somatosensory square dance system to address existing privacy, noise, and space shortage conflicts as well as to provide adequate training. In previous studies, dance-based aerobic exercise was proved to improve indices of falling risk and lower body function in older persons; it was also proved to provide psychological benefits [5,30,31].

The establishment of the Laban Elated Square Dance was in line with the previously mentioned aspects. The Laban Elated Square Dance was professionally choreographed for balance training of older persons, and it greatly addresses the problem of poor professional guidance. Most participants stated that the lack of square dance fitness guidance is one of the greatest deficits in square dancing, which was a hidden issue.

Square dance movement involves many different aspects, and the participants suggested that only the music beat should be considered in the movements while ignoring the physiological conditions of older people. Square dancing causes joint injuries; however, in previous studies, the square dance choreography was not optimized to prevent this. The Laban Elated Square Dance system meets the physical and psychological needs of participants, addresses current issues, and can be provided to any square dance group. Previous studies showed that video games can improve physical and psychological health and are beneficial to the quality of life of older people [32-36].

This study assessed the usability of a somatosensory square dance system based on the subjective experience of participants as described in interviews, a satisfaction scale questionnaire with psychological, physiological, and relaxation sections, and system design and acceptance by the participants. The results showed that the overall evaluation and acceptance of the somatosensory system was generally positive. The satisfaction scale scores showed that the overall evaluations of the three sections were close to satisfactory. Participants’ relaxation scores ranged between satisfied and very satisfied. The results were consistent with Beard’s interpretation of needs: participation in leisure activities can help people relax and can relieve their daily life pressure. This study also supported the ability of the Kinect-based somatosensory technology equipment mentioned in the literature review to improve rehabilitation and balance [26-28].

The results of the evaluation of the system were encouraging. The overall evaluation of the system was positive; however, for individuals with different exercise styles, the experiences of this system by the target user groups were different. Middle-aged and older people who continuously participated in traditional outdoor square dance repeatedly mentioned the words “exercise,” “chat,” “interact,” and “communicate;” they considered the Laban Elated Square Dance system to be an auxiliary tool. For those who do not understand or participate in outdoor square dance, this system may provide a platform for them to overcome shyness and fear of shame and to enjoy the exercise and entertainment functions of square dance in a private space.

### Limitations

There are limitations of this study. The interaction interface designed in the somatosensory system could be enhanced and made more attractive for older people to use. The participants in the system verification experiment were all recruited from one university, which may result in geographical bias. The interview outline is specifically designed for the participants to provide feedback about their experience of the system, which is a potential limitation of this research.

Although a somatosensory system cannot completely replace square dance, this newly designed technology could help spread Chinese square dance culture; thus, this unique Chinese cultural experience may become international, and people in the Western World can experience Chinese square dance in a somatosensory way.

### Conclusions

We developed a novel somatosensory square dance system as an indoor dancing and fitness training tool for older people that integrates the motivational attributes of video games with functional sensory tracking. Usability tests (interviews and a questionnaire) with a small sample of older and younger people demonstrated the evaluation, acceptance, and suggestions for improvement of the system. The results indicate that the system can be used as an indoor square dance fitness tool for exercise and practice in the future. It provides a platform for people to overcome shyness and fear of shame and to enjoy the exercise and entertainment functions of square dancing in a private space. With the professional guidance within the system, dancers can receive targeted training indoors and can communicate more with family members. Moreover, with the application of the system, competition for outdoor space and noise conflicts will be alleviated. Data from the motion-recorded video can be transmitted to physicians for motion analysis and disease prediction. Our goal was to produce more interactive and social functions for square dancers and contribute to family medical care. Different feature levels will be considered in the next stage, such as selection of exercises and difficulty level settings; users’ social needs and a larger sample size will also be explored in subsequent stages. Alternative software, such as iClone Motion and FXhome Action Pro, can be used as to capture users’ motion.

## Abbreviations

LMA: Laban Movement Analysis
